# Effects of yoga breathing exercises on pulmonary function in patients with Duchenne muscular dystrophy: an exploratory analysis*, **

**DOI:** 10.1590/S1806-37132014000200005

**Published:** 2014

**Authors:** Marcos Rojo Rodrigues, Celso Ricardo Fernandes Carvalho, Danilo Forghieri Santaella, Geraldo Lorenzi-Filho, Suely Kazue Nagahashi Marie

**Affiliations:** Sports Center, University of São Paulo, São Paulo, Brazil; Department of Physical Therapy, University of São Paulo School of Medicine, São Paulo, Brazil; Sports Center, University of São Paulo, São Paulo, Brazil; Department of Pulmonology, Heart Institute, University of São Paulo School of Medicine Hospital das Clínicas , São Paulo, Brazil; Department of Neurology, University of São Paulo School of Medicine, São Paulo, Brazil

**Keywords:** Respiratory therapy, Forced expiratory volume, Vital capacity, Muscular dystrophy, Duchenne, Complementary therapies

## Abstract

**OBJECTIVE:**

Duchenne muscular dystrophy (DMD) is the most common form of muscular dystrophy in children, and children with DMD die prematurely because of respiratory failure. We sought to determine the efficacy and safety of yoga breathing exercises, as well as the effects of those exercises on respiratory function, in such children.

**METHODS:**

This was a prospective open-label study of patients with a confirmed diagnosis of DMD, recruited from among those followed at the neurology outpatient clinic of a university hospital in the city of São Paulo, Brazil. Participants were taught how to perform hatha yoga breathing exercises and were instructed to perform the exercises three times a day for 10 months.

**RESULTS:**

Of the 76 patients who entered the study, 35 dropped out and 15 were unable to perform the breathing exercises, 26 having therefore completed the study (mean age, 9.5 ± 2.3 years; body mass index, 18.2 ± 3.8 kg/m^2)^. The yoga breathing exercises resulted in a significant increase in FVC (% of predicted: 82.3 ± 18.6% at baseline vs. 90.3 ± 22.5% at 10 months later; p = 0.02) and FEV_1_ (% of predicted: 83.8 ± 16.6% at baseline vs. 90.1 ± 17.4% at 10 months later; p = 0.04).

**CONCLUSIONS:**

Yoga breathing exercises can improve pulmonary function in patients with DMD.

## Introduction

Duchenne muscular dystrophy (DMD) is the most common form of muscular dystrophy in children, occurring in 1 of every 3,000-3,500 male births.^(^ ^1^ ^,^ ^2^ ^)^ There is evidence that corticosteroid therapy has beneficial effects on the health and quality of life of patients with DMD and can decrease the requirement for nocturnal ventilation in such patients.^(^ ^3^ ^)^ As a result of dystrophin deficiency, patients with DMD (including those who receive optimal treatment) experience a progressive loss of muscle fiber that eventually affects the respiratory muscles.^(^ ^2^ ^)^ The resulting respiratory failure is the most common cause of premature death in patients with DMD.^(^ ^1^ ^,^ ^4^ ^-^ ^6^ ^)^

Respiratory muscles are progressively compromised in children with DMD, the expiratory muscles being the most commonly affected.^(^ ^7^ ^)^ Even short periods of physical inactivity can contribute to muscle weakness and reduced respiratory capacity.^(^ ^8^ ^-^ ^10^ ^)^ There is evidence that breathing exercises can improve respiratory function in patients with DMD.^(^ ^11^ ^,^ ^12^ ^)^ Hatha yoga is a broad philosophy that encompasses a series of breathing exercises aimed at improving the health of its practitioners. The objective of the present study was to determine whether a 10-month program of yoga breathing exercises that recruit inspiratory and expiratory muscles is safe for children with DMD and can improve their respiratory function.

## Methods

The study sample consisted of consecutive patients treated at the neurology outpatient clinic of a university hospital in the city of São Paulo, Brazil. The inclusion criteria were as follows: having been diagnosed with DMD (as confirmed by molecular assessment of the skeletal muscle); being in the 6-14 year age bracket; and using corticosteroids regularly for at least 3 months. Severely ill children who were unable to perform the breathing exercises were excluded from the study. The study protocol was approved by the local research ethics committee, and the parents or legal guardians of all participants gave written informed consent.

The present study was conducted over a 10-month period, with clinical evaluations being performed in the morning at study entry (baseline) and at study termination. During the study period, the patients returned for clinical evaluations at regular intervals (of 1-2 months).

All children were individually taught how to perform the breathing exercises while sitting in a quiet room, practicing each exercise until they were able to perform it without supervision. The children were taught a new exercise at each clinical evaluation. The first breathing exercise that they were taught was kapalabhati, consisting of nasal exhalations produced by fast, vigorous contraction of the abdominal and pelvic muscles, followed by passive inhalations produced by relaxation of the recruited muscles. At 3 months after study entry, the children were taught another breathing exercise, which is known as uddiyana and consists of apnea after forced expiration, followed by thoracic expansion (achieved without inhalation) and voluntary glottic closure. At 6 months after study entry, the children were taught yet another breathing exercise, which is known as agnisara and consists of maximal contraction followed by abdominal projection during apnea after forced expiration. The participants were instructed to perform this sequence of exercises three times a day, every day, as follows: three series of 120 repetitions for kapalabhati; three 10-s repetitions for uddiyana; and three series of five movements for agnisara. Caregivers kept a diary, in which they marked an "x" every time the children performed the home exercises. The diaries were returned to the researchers on a monthly basis. We included only those children whose adherence to the exercise program was at least 75%.

Spirometry was performed with a dry bellows spirometer (Koko Spirometer; PDS Instrumentation, Inc., Louisville, CO, USA) in accordance with the American Thoracic Society/European Respiratory Society Task Force standards for lung function testing.^(^ ^13^ ^)^ We measured FEV_1_ and FVC, predicted normal values being determined by the use of validated equations.^(^ ^14^ ^)^ We measured MEP and MIP at the mouth using a portable spirometer (microQuark; Cosmed, Rome, Italy) under static conditions, in accordance with a validated method.^(^ ^15^ ^)^ We measured MEP at TLC and MIP at functional residual capacity, the highest of three valid measurements being recorded. Before these measurements were obtained, patients were allowed to make at least three attempts. The results are expressed as relative values (percentages of the predicted values for age).

Normality was tested with the Kolmogorov-Smirnov test. The Student's t-test for repeated measures (baseline vs. 10 months after study entry) was used in order to evaluate the effects of the intervention on all physiological variables. The level of significance was set at p < 0.05. The results were analyzed with the Statistical Package for the Social Sciences, version 16.0 (SPSS Inc., Chicago, IL, USA).

## Results

**Figure 1:**
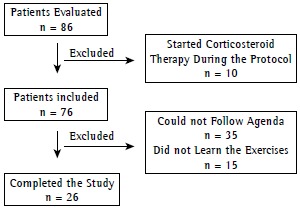
Flowchart of the study.

A total of 86 patients with a confirmed diagnosis of DMD were initially considered for inclusion. Of those, 10 were ineligible because they were not receiving corticosteroids. Therefore, 76 patients entered the study. Of those, 35 dropped out and 15 were unable to perform the exercises, being therefore excluded from the study. Many of the patients who dropped out lived in cities that are far from where the present study was conducted and were therefore unable to attend the scheduled clinical evaluations (Figure 1). The demographic characteristics and pulmonary function test results of the 76 patients who entered the study are presented in Table 1.

**Table 1:** Demographic characteristics and pulmonary function test results of the Duchenne muscular dystrophy patients studied.a

Variable	Patients
	(n = 26)
Demographic data	
Age, years	9.5 ± 2.2
Weight, kg	31.6 ± 11.4
Height, cm	130.3 ± 13+8
Body mass index, kg/m^2^	18.2 ± 3.8
Pulmonary function test results	
FVC, % of predicted	82.9 ± 16.8
FEV_1_, % of predicted	84.3 ± 16.0
MEP, % of predicted	63.9 ± 27.6
MIP, % of predicted	41.4 ± 13.7

a Data expressed as mean ± SD

**Figure 2:**
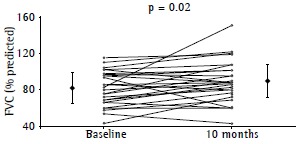
FVC (% of predicted) at baseline and at 10 months after study entry. Note that FVC was significantly higher at 10 months after study entry. Rhomboids represent mean ± SD.

**Figure 3:**
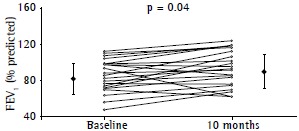
FEV1 (% of predicted) at baseline and at 10 months after study entry. Note that FEV1 was significantly higher at 10 months after study entry. Rhomboids represent mean ± SD.

There were no significant differences between MEP at baseline and MEP at 10 months after study entry (63.9 ± 27.6% of predicted vs. 66.8 ± 27.6% of predicted; p > 0.05) or between MIP at baseline and MIP at 10 months after study entry (41.4 ± 13.7% of predicted vs. 43.7 ± 12.8% of predicted; p > 0.05). However, FVC at baseline was significantly lower than FVC at 10 months after study entry (82.3 ± 18.6% of predicted vs. 90.3 ± 22.5% of predicted; p = 0.02; Figure 2). Likewise, FEV_1_ at baseline was significantly lower than FEV_1_ at 10 months after study entry (83.8 ± 16.6% of predicted vs. 90.1 ± 17.4% of predicted; p = 0.04; Figure 3).

## Discussion

The present study evaluated children with DMD receiving optimal medical therapy with corticosteroids. In children with DMD, respiratory function is expected to deteriorate over time, at a rate of 5% per year.^(^ ^7^ ^)^ The novel findings of the present study include the fact that a large proportion of patients with DMD (82.6%) were able to learn how to perform yoga breathing exercises and the fact that pulmonary function can improve over a 10-month period, as evidenced by significant improvements in FEV_1_ and FVC.

Over the course of DMD, absolute values of lung capacity initially increase, then reach a plateau, and finally decrease. In contrast, relative values (proportional to the values predicted for age and gender) of all pulmonary function variables show a constant decline after the age of 5 years. ^(^ ^12^ ^,^ ^16^ ^,^ ^17^ ^)^ For instance, respiratory capacity decreases by approximately 5% per year.^(^ ^12^ ^)^ Because DMD is a relatively rare and progressive disease with an expected survival of 16-19 years,^(^ ^18^ ^)^ the best evidence for standard of care and new treatments is primarily from observational studies. One group of authors^(^ ^19^ ^)^ evaluated 40 DMD patients who were treated with corticosteroids in comparison with 34 who were not and concluded that lung function stabilization constitutes evidence of the beneficial effect of corticosteroids on DMD. Similar findings were recently reported for patients in Brazil. In our study, we included only DMD patients who were using corticosteroids regularly and used predicted values of pulmonary function as primary endpoints. In fact, it has been demonstrated that FVC (in % of predicted) decreases by approximately 4% per year in DMD patients.^(^ ^20^ ^)^ In our patients, who participated in a 10-month yoga exercise program, lung function stabilized (which is, in and of itself, a positive effect) and there were increases in FEV_1_ and FVC (both in % of predicted). Therefore, although our study had no control group, the results should be interpreted as evidence that yoga breathing exercises have an additive positive effect on the lung function of DMD patients undergoing conventional treatment.

One study showed that respiratory muscle training can be beneficial to patients with DMD. ^(^ ^21^ ^)^ Despite the lack of studies addressing the use of yoga breathing exercises as complementary therapy for DMD patients, there have been clinical respiratory studies of the topic, although the results have been controversial. In a recent review of the effects of breathing exercises on COPD,^(^ ^22^ ^)^ such exercises were found to be effective in increasing the six-minute walk distance. In contrast, another review showed that yoga had no effect on cardiorespiratory variables in asthma patients.^(^ ^23^ ^)^ However, those two reviews evaluated the effects of yoga in adults. In children with asthma, FEV_1_ was significantly increased after yoga training,^(^ ^24^ ^)^ which included yoga postures and breathing exercises. To our knowledge, the present study is the first to address and confirm the safety and efficacy of yoga breathing exercises in children with DMD. The yoga breathing exercises used in the present study are unique and were chosen because children can learn them after only a few sessions and subsequently practice them by themselves without the need for additional equipment or continuous supervision, the exercise program being therefore accessible to a large number of patients. During the exercise known as kapalabhati, abdominal muscles are kept active, working rapidly and vigorously as expiratory muscles. Exhalation occurs below functional residual capacity and therefore becomes active, whereas inhalation becomes passive. This is in contrast with what occurs physiologically (inhalation being active and exhalation being passive). Kapalabhati therefore allows expiratory muscle training while the inspiratory muscles are at rest. Kapalabhati was the first exercise that the children were taught, being therefore the most practiced exercise and the exercise that probably contributed the most to the results obtained. Both uddiyana and agnisara have conditioning effects on the inspiratory muscles, the former focusing primarily on the intercostal respiratory muscles and the latter focusing primarily on the diaphragm.

Our study has strengths and limitations. The primary objective of the study was to show that yoga breathing exercises can be performed by patients with DMD. This hypothesis had to be carefully tested, particularly because DMD is a progressive, genetic disease, and breathing exercises could have deleterious effects on such patients (i.e., effects similar to those of overtraining in healthy individuals). In fact, the dropout rate was high in our sample. Although this might be due to difficulty breathing during the exercises, we believe that it was probably due to the fact that those patients were unable to attend the exercise sessions; that is, they had no means of transportation. In addition, only a small proportion of patients dropped out because they were unable to perform spirometry (either because they had respiratory problems or because they were unable to learn how to perform the exercises). Furthermore, there were no significant differences among the patients regarding any of the respiratory variables studied. Our study therefore showed that yoga breathing exercises are feasible, are harmless, and can actually improve respiratory function. Although our results clearly show that pulmonary function improved in terms of the predicted values-meaning that the increase was observed compared to each subject-randomized controlled studies are needed in order to confirm that. It is of note that DMD is a rare, fatal disease, and the best evidence for any form of treatment is from observational studies.

In conclusion, yoga exercises are feasible and can improve lung function in children with DMD. Further studies are needed in order to determine whether yoga exercises can improve quality of life and reduce the number of hospital admissions in such patients.

## References

[1] Simonds AK (2002). Respiratory complications of the muscular
dystrophy. Sem Resp Crit Care Med.

[2] Machado DL, Silva EC, Resende MB, Carvalho CR, Zanoteli E, Reed UC (2012). Lung function monitoring in patients with duchenne
muscular dystrophy on steroid therapy. BMC Res Notes.

[3] Bach JR, Martinez D, Saulat B (2010). Duchenne muscular dystrophy: the effect of
glucocorticoids on ventilator use and ambulation. Am J Phys Med Rehabil.

[4] Braun NM, Arora NS, Rochester DF (1983). Respiratory muscle and pulmonary function in
polymyositis and other proximal myopathies. Thorax.

[5] Vincken WG, Elleker MG, Cosio MG (1987). Flow-volume loop changes reflecting respiratory muscle
weakness in clinical neuromuscular disorders. Am J Med.

[6] Dolmage TE, Avendano MA, Goldstein RS (1992). Respiratory function during wake-fulness and sleep among
survivors of respiratory and non-respiratory poliomyelitis. Eur Respir J.

[7] Tangsrud S, Petersen IL, Lødrup Carlsen KC, Carlsen KH (2001). Lung function in children with Duchenne's muscular
dystrophy. Respir Med.

[8] De Troyer A, Borenstein S, Cordier R (1980). Analysis of lung restriction in patients with
respiratory muscle weakness. Thorax.

[9] Mier-Jedrzejowicz A, Brophy C, Green M (1988). Respiratory muscle weakness during respiratory tract
infections. Am Rev Respir Dis.

[10] Bach JR, Rajaraman R, Ballanger F, Tzeng AC, Ishikawa Y, Kulessa R, Bansal T (1998). Neuromuscular ventilator insufficiency: effect of home
mechanical ventilation v oxygen therapy on pneumonia and hospitalization
rates. Am J Phys Med Rehabil.

[11] Matecki S, Topin N, Hayot M, Rivier F, Echenne B, Prefaut C (2001). A standardized method for the evaluation of respiratory
muscle endurance in patients with Duchnne muscular dystrophy. Neuromuscul Disord.

[12] Topin N, Matecki S, Le Bris S, Rivier F, Echenne B, Prefaut C (2002). Dose-dependent effect of individualized respiratory
muscle training in children with Duchenne muscular dystrophy. Neuromuscul Disord.

[13] Miller MR, Hankinson J, Brusasco F, Burgos F, Casaburi R, Coates A (2005). Standardisation of spirometry. Eur Respir J.

[14] Duarte AA, Pereira CA, Barreto SC (2007). Validation of new brazilian predicted values for forced
spirometry in caucasians and comparison with predicted values obtained using other
reference equations. J Bras Pneumol.

[15] Black LF, Hyatt RE (1969). Maximal respiratory pressures: normal values and
relationship to age and sex. Am Rev Respir Dis.

[16] Hahn A, Bach JR, Delaubier A, Renardel-Irani A, Guillou C, Rideau Y (1997). Clinical implications of maximal respiratory pressure
determinations for individuals with Duchenne muscular dystrophy. Arch Phys Med Rehabil.

[17] Griggs RC, Donohoe KM, Utell MJ, Goldblatt D, Moxley 3rd RT (1981). Evaluation of pulmonary function in neuromuscular
disease. Arch Neurol.

[18] Bach JR, Ishikawa Y, Kim H (1997). Prevention of pulmonary morbidity for patients with
Duchenne muscular dystrophy. Chest.

[19] Biggar WD, Harris VA, Eliasoph L, Alman B (2006). Long-term benefits of deflazacort treatment for boys
with Duchenne muscular dystrophy in their second decade. Neuromuscul Disord.

[20] Khirani S, Ramirez A, Aubertin G, Boulé M, Chemouny C, Forin V (2013). Respiratory muscle decline in duchenne muscular
dystrophy. Pediatr Pulmonol.

[21] Topin N, Matecki S, Le Bris S, Rivier F, Echenne B, C Prefaut C (2002). Dose-dependent effect of individualized respiratory
muscle training in children with Duchenne muscular dystrophy. Neuromuscul Disord.

[22] Holland AE, Hill CJ, Jones AY, McDonald CF (2012). Breathing exercises for chronic obstructive pulmonary
disease. Cochrane Database Syst Rev.

[23] Posadzki P, Ernst E (2011). Yoga for asthma: A systematic review of randomized
clinical trials. J Asthma.

[24] Field T (2012). Exercise research on children and
adolescents. Complement Ther Clin Pract.

