# Supplementary material for: Effects of yoga breathing exercises on pulmonary function in patients with Duchenne muscular dystrophy: an exploratory analysis
Source: J Bras Pneumol. 2014 Mar-Apr;40(2):128–33. doi: 10.1590/S1806-37132014000200005 (PMC4083644; doi:10.1590/S1806-37132014000200005)
Supplement: Translation

# Translation: Efeitos de exercícios respiratórios de ioga na função pulmonar de pacientes com distrofia muscular de Duchenne: uma análise exploratória*

**Authors:** Marcos Rojo Rodrigues, Celso Ricardo Fernandes Carvalho, Danilo Forghieri Santaella, Geraldo Lorenzi-Filho, Suely Kazue Nagahashi Marie

**Affiliations:** Centro de Práticas Esportivas, Universidade de São Paulo, São Paulo (SP) Brasil; Departamento de Fisioterapia, Faculdade de Medicina da Universidade de São Paulo, São Paulo (SP) Brasil; Centro de Práticas Esportivas, Universidade de São Paulo, São Paulo (SP) Brasil; Departamento de Pneumologia, Instituto do Coração, Hospital das Clínicas, Faculdade de Medicina da Universidade de São Paulo, São Paulo (SP) Brasil; Professora Associada. Departamento de Neurologia, Faculdade de Medicina da Universidade de São Paulo, São Paulo (SP) Brasil

**Keywords:** Terapia respiratória, Volume expiratório forçado, Capacidade vital, Distrofia muscular de Duchenne, Terapias complementares

## Abstract

**OBJETIVO:**

A distrofia muscular de Duchenne (DMD) é a forma mais comum de distrofia muscular em crianças, e crianças com DMD morrem prematuramente por causa de insuficiência respiratória. Analisamos a eficácia e segurança de exercícios respiratórios de ioga nessas crianças, bem como os efeitos desses exercícios em sua função respiratória.

**MÉTODOS:**

Estudo prospectivo aberto envolvendo pacientes com diagnóstico confirmado de DMD recrutados no ambulatório de neurologia de um hospital universitário em São Paulo (SP). Os participantes aprenderam exercícios respiratórios de hatha ioga e foram instruídos a praticá-los três vezes ao dia durante 10 meses.

**RESULTADOS:**

Dos 76 pacientes incluídos no estudo, 35 o abandonaram e 15 não conseguiram realizar os exercícios respiratórios, de modo que 26 pacientes completaram o estudo (média de idade: 9,5 ± 2,3 anos; índice de massa corporal: 18,2 ± 3,8 kg/m^2)^. Os exercícios respiratórios de ioga resultaram em um aumento significativo da CVF em porcentagem do previsto (82,3 ± 18,6% antes do início do programa de exercícios vs. 90,3 ± 22,5% 10 meses depois; p = 0,02) e do VEF_1_ em porcentagem do previsto (83,8 ± 16,6% antes do início do programa de exercícios vs. 90,1 ± 17,4% 10 meses depois; p = 0,04).

**CONCLUSÕES:**

Os exercícios respiratórios de ioga podem melhorar a função pulmonar de pacientes com DMD.

## Introdução

A distrofia muscular de Duchenne (DMD) é a forma mais comum de distrofia muscular em crianças; de cada 3.000-3.500 recém-nascidos do sexo masculino, 1 é afetado.^(^ ^1^ ^,^ ^2^ ^)^ Há evidências de que a corticoterapia tem efeitos benéficos sobre a saúde e a qualidade de vida de pacientes com DMD e pode diminuir a necessidade de ventilação noturna nesses pacientes.^(^ ^3^ ^)^ Como resultado da deficiência de distrofina, pacientes com DMD (inclusive aqueles que recebem o melhor tratamento) sofrem perda progressiva de fibras musculares, que acaba por afetar os músculos respiratórios.^(^ ^2^ ^)^ A insuficiência respiratória resultante é a causa mais comum de morte prematura em pacientes com DMD.^(^ ^1^ ^,^ ^4^ ^-^ ^6^ ^)^

Os músculos respiratórios são progressivamente afetados em crianças com DMD, sendo os músculos expiratórios os mais afetados.^(^ ^7^ ^)^ Mesmo curtos períodos de inatividade física podem contribuir para que ocorra fraqueza muscular e redução da capacidade respiratória.^(^ ^8^ ^-^ ^10^ ^)^ Há evidências de que exercícios respiratórios podem melhorar a função respiratória em pacientes com DMD. ^(^ ^11^ ^,^ ^12^ ^)^ A hatha ioga é uma filosofia ampla que engloba uma série de exercícios respiratórios cujo objetivo é melhorar a saúde de seus praticantes. O objetivo do presente estudo foi determinar se um programa de 10 meses de exercícios respiratórios de ioga que recrutam a musculatura inspiratória e expiratória é seguro para crianças com DMD e pode melhorar sua função respiratória.

## Métodos

A amostra foi composta por pacientes consecutivos tratados no ambulatório de neurologia de um hospital universitário na cidade de São Paulo (SP). Os critérios de inclusão foram os seguintes: diagnóstico de DMD (confirmado por avaliação molecular do músculo esquelético); faixa etária de 6-14 anos e uso regular de corticosteroides durante pelo menos 3 meses. Crianças gravemente doentes que não conseguiram realizar os exercícios respiratórios foram excluídas do estudo. O protocolo do estudo foi aprovado pelo comitê de ética em pesquisa da instituição, e todos os pais ou responsáveis assinaram um termo de consentimento livre e esclarecido.

O presente estudo foi realizado durante um período de 10 meses, com avaliações clínicas realizadas no período da manhã no início do estudo (antes do programa de exercícios) e no fim do estudo. Durante o estudo, os pacientes retornaram para avaliações clínicas a intervalos regulares (de 1-2 meses).

As crianças foram ensinadas a realizar os exercícios respiratórios individualmente, sentadas em uma sala silenciosa, praticando cada exercício até que fossem capazes de realizá-lo sem supervisão. As crianças aprenderam um exercício diferente a cada avaliação clínica. O primeiro exercício respiratório que lhes foi ensinado foi *kapalabhati*, que consiste em expirações nasais produzidas pela contração rápida e vigorosa dos músculos abdominais e pélvicos, seguidas de inspirações passivas produzidas pelo relaxamento dos músculos recrutados. Três meses após o início do estudo, as crianças foram ensinadas a realizar outro exercício respiratório, denominado *uddiyana*, que consiste em apneia após expiração forçada, seguida de expansão torácica (conseguida sem inspiração) e fechamento glótico voluntário. Seis meses após o início do estudo, as crianças foram ensinadas a realizar mais um exercício respiratório, denominado *agnisara*, que consiste em contração máxima seguida de projeção abdominal durante a apneia após expiração forçada. Os participantes foram instruídos a realizar essa sequência de exercícios três vezes por dia, todos os dias, da seguinte forma: três séries de 120 repetições de *kapalabhati*; três repetições de 10 s de *uddiyana* e três séries de cinco movimentos de *agnisara*. Em um diário, os cuidadores marcavam um "x" sempre que as crianças realizavam os exercícios de casa. Os diários eram devolvidos aos pesquisadores mensalmente. Foram incluídas apenas as crianças cuja adesão ao programa de exercícios foi de pelo menos 75%.

A espirometria foi realizada com um espirômetro seco de fole (Espirômetro Koko; PDS Instrumentation, Inc., Louisville, CO, EUA) conforme os padrões para testes de função pulmonar estabelecidos pela Força-Tarefa da *American Thoracic Society*/*European Respiratory Society*.^(^ ^13^ ^)^ Foram medidos o VEF_1_ e a CVF, e os valores normais previstos foram determinados por meio de equações validadas.^(^ ^14^ ^)^ A PEmáx e a PImáx foram medidas na boca com um espirômetro portátil (microQuark; Cosmed, Roma, Itália) em condições estáticas, de acordo com um método validado.^(^ ^15^ ^)^ A PEmáx foi medida em CPT e a PImáx foi medida em capacidade residual funcional, sendo registrada a maior de três medidas válidas. Antes dessas medições, os pacientes podiam fazer pelo menos três tentativas. Os resultados são expressos na forma de valores relativos (porcentagens dos valores previstos para a idade).

A normalidade foi testada por meio do teste de Kolmogorov-Smirnov. O teste t de Student para medidas repetidas (antes do início do estudo vs. 10 meses depois) foi usado para avaliar os efeitos da intervenção sobre todas as variáveis fisiológicas. O nível de significância adotado foi de p < 0,05. Os resultados foram analisados por meio do programa *Statistical Package for the Social Sciences*, versão 16.0 (SPSS Inc., Chicago, IL, EUA).

## Resultados

Considerou-se inicialmente a inclusão de 86 pacientes com diagnóstico confirmado de DMD. Desses 86 pacientes, 10 não estavam aptos a participar, pois não estavam recebendo tratamento com corticosteroides. Portanto, 76 pacientes entraram no estudo. Desses 76 pacientes, 35 abandonaram o estudo e 15 não conseguiram realizar os exercícios, sendo, portanto, excluídos do estudo. Muitos dos pacientes que abandonaram o estudo viviam em cidades distantes de onde o presente estudo foi realizado e, portanto, não conseguiram comparecer às avaliações clínicas agendadas (Figura 1). As características demográficas e os resultados dos testes de função pulmonar dos 76 pacientes que entraram no estudo são apresentados na Tabela 1.

**Figura 1:** Fluxograma do estudo.

**Tabela 1:** Características demográficas e resultados dos testes de função pulmonar dos pacientes com distrofia muscular de Duchenne.a

Variáveis	Pacientes
	(n = 26)
Dados demográficos	
Idade, anos	9,5 ± 2,2
Peso, kg	31,6 ± 11,4
Estatura, cm	130,3 ± 13+8
Índice de massa corporal, kg/m^2^	18,2 ± 3,8
Resultados dos testes de função pulmonar	
CVF, % do previsto	82,9 ± 16,8
VEF_1_, % do previsto	84,3 ± 16,0
PEmáx, % do previsto	63,9 ± 27,6
PImáx, % do previsto	41,4 ± 13,7

a Dados expressos na forma de média ± dp.

Não houve diferenças significativas entre a PEmáx medida no início do estudo e aquela medida 10 meses depois (63,9 ± 27,6% do previsto vs. 66,8 ± 27,6% do previsto; p > 0,05) ou entre a PImáx medida no início do estudo e aquela medida 10 meses depois (41,4 ± 13,7% do previsto vs. 43,7 ± 12,8% do previsto; p > 0,05). No entanto, a CVF medida no início do estudo foi significativamente menor do que aquela medida 10 meses depois (82,3 ± 18,6% do previsto vs. 90,3 ± 22,5% do previsto; p = 0,02; Figura 2). Da mesma forma, o VEF_1_ medido no início do estudo foi significativamente menor do que aquele medido 10 meses depois (83,8 ± 16,6% do previsto vs. 90,1 ± 17,4% do previsto; p = 0,04; Figura 3).

**Figura 2:** CVF (% do previsto) antes do início do estudo e 10 meses depois. Nota-se que a CVF estava significativamente maior 10 meses depois. Os losangos representam média ± dp.

**Figura 3:** VEF1 (% do previsto) antes do início do estudo e 10 meses depois. Nota-se que o VEF1 estava significativamente maior 10 meses depois. Os losangos representam média ± dp.

## Discussão

O presente estudo avaliou crianças com DMD que recebiam o melhor tratamento médico, com corticosteroides. Em crianças com DMD, espera-se que a função respiratória se deteriore com o tempo, a uma taxa de 5% por ano.^(^ ^7^ ^)^ Dentre os achados inéditos do presente estudo estão o fato de que uma grande porcentagem de pacientes com DMD (82,6%) aprendeu a realizar exercícios respiratórios de ioga e o fato de que a função pulmonar pode melhorar ao longo de um período de 10 meses, conforme evidenciou a melhora significativa do VEF_1_ e da CVF.

Durante a DMD, os valores absolutos de capacidade pulmonar inicialmente aumentam; em seguida, estabilizam-se e, finalmente, diminuem. Por outro lado, os valores relativos (proporcionais aos valores previstos para a idade e o gênero) de todas as variáveis de função pulmonar diminuem constantemente após os 5 anos de idade.^(^ ^12^ ^,^ ^16^ ^,^ ^17^ ^)^ Por exemplo, a capacidade respiratória diminui cerca de 5% por ano.^(^ ^12^ ^)^ Como a DMD é uma doença relativamente rara e progressiva a qual se atribui uma expectativa de sobrevida de 16-19 anos,^(^ ^18^ ^)^ as melhores evidências de padrão de tratamento e novos tratamentos provêm principalmente de estudos observacionais. Um grupo de autores^(^ ^19^ ^)^ avaliou 40 pacientes com DMD tratados com corticosteroides em comparação com 34 que não o foram e concluiu que a estabilização da função pulmonar é evidência do efeito benéfico dos corticosteroides sobre a DMD. Achados semelhantes foram recentemente descritos em pacientes no Brasil. Em nosso estudo, incluímos apenas pacientes com DMD que usavam corticosteroides regularmente e usamos valores previstos de função pulmonar como desfechos primários. De fato, demonstrou-se que a CVF (em % do previsto) diminui cerca de 4% por ano em pacientes com DMD.^(^ ^20^ ^)^ Em nossos pacientes, que participaram de um programa de 10 meses de exercícios de ioga, a função pulmonar se estabilizou (o que por si só é um efeito positivo) e tanto o VEF_1_ como a CVF (ambos em % do previsto) aumentaram. Portanto, embora nosso estudo não tenha tido um grupo controle, os resultados devem ser interpretados como evidência de que exercícios respiratórios de ioga têm efeito aditivo positivo sobre a função pulmonar de pacientes com DMD em tratamento convencional.

Um estudo mostrou que o treinamento muscular respiratório pode ser benéfico para pacientes com DMD.^(^ ^21^ ^)^ Apesar da escassez de estudos sobre o uso de exercícios respiratórios de ioga como terapia complementar para pacientes com DMD, já houve estudos clínicos respiratórios sobre o assunto, embora os resultados tenham sido controversos. Em uma recente revisão dos efeitos de exercícios respiratórios sobre a DPOC,^(^ ^22^ ^)^ constatou-se que tais exercícios são eficazes em aumentar a distância percorrida no teste de caminhada de seis minutos. Por outro lado, uma revisão mostrou que a ioga não teve efeito sobre variáveis cardiorrespiratórias em pacientes com asma.^(^ ^23^ ^)^ No entanto, essas duas revisões avaliaram os efeitos da ioga em adultos. Em crianças com asma, o VEF_1_ aumentou significativamente após o treinamento de ioga,^(^ ^24^ ^)^ que incluiu posturas de ioga e exercícios respiratórios. Até onde sabemos, o presente estudo é o primeiro a examinar e confirmar a segurança e a eficácia de exercícios respiratórios de ioga em crianças com DMD. Os exercícios respiratórios de ioga usados no presente estudo são únicos e foram escolhidos porque as crianças podem aprendê-los depois de apenas algumas sessões e, posteriormente, praticá-los sozinhas, sem a necessidade de equipamento adicional ou de supervisão contínua; o programa de exercícios é, portanto, acessível a um grande número de pacientes. Durante o exercício conhecido como *kapalabhati*, os músculos abdominais mantêm-se ativos, trabalhando rápida e vigorosamente como músculos expiratórios. A expiração ocorre abaixo da capacidade residual funcional e, portanto, torna-se ativa, ao passo que a inspiração se torna passiva. Isso está em contraste com o que ocorre fisiologicamente (isto é, inspiração ativa e expiração passiva). Portanto, *kapalabhati* permite o treinamento dos músculos expiratórios enquanto os músculos inspiratórios permanecem em repouso. *Kapalabhati* foi o primeiro exercício ensinado às crianças, sendo, portanto, o exercício mais praticado e o que provavelmente mais contribuiu para os resultados obtidos. Tanto *uddiyana* como *agnisara* condicionam a musculatura inspiratória, sendo este voltado principalmente ao diafragma e aquele aos músculos respiratórios intercostais.

Nosso estudo tem pontos fortes e limitações. O objetivo primário do estudo foi mostrar que exercícios respiratórios de ioga podem ser realizados por pacientes com DMD. Essa hipótese tinha de ser cuidadosamente testada, especialmente porque a DMD é uma doença genética, progressiva, e exercícios respiratórios poderiam ter efeitos deletérios nesses pacientes (isto é, efeitos semelhantes aos do supertreinamento em indivíduos saudáveis). De fato, a taxa de abandono foi alta em nossa amostra. Embora seja possível que isso tenha ocorrido em virtude de dificuldade para respirar durante os exercícios, acreditamos que tenha provavelmente ocorrido porque esses pacientes não puderam comparecer às sessões de exercício, isto é, não tinham meios para se transportar até o local. Além disso, apenas uma pequena porcentagem de pacientes abandonou o estudo porque não conseguiu realizar a espirometria (quer porque tinham problemas respiratórios, quer porque não aprenderam a realizar os exercícios). Ademais, não houve diferenças significativas entre os pacientes quanto às variáveis respiratórias estudadas. Portanto, nosso estudo mostrou que exercícios respiratórios de ioga são exequíveis, inofensivos e podem realmente melhorar a função respiratória. Embora nossos resultados mostrem claramente que a função pulmonar melhorou no que tange aos valores previstos, isto é, o aumento foi observado em relação a cada paciente, são necessários estudos controlados randomizados para confirmar isso. É digno de nota que a DMD é uma doença rara e fatal, e as melhores evidências de quaisquer formas de tratamento provêm de estudos observacionais.

Em suma, os exercícios de ioga são exequíveis e podem melhorar a função pulmonar em crianças com DMD. Mais estudos são necessários para determinar se exercícios de ioga podem melhorar a qualidade de vida e reduzir o número de internações hospitalares em tais pacientes.
